# Age-Dependent Chronic Lung Injury and Pulmonary Fibrosis following Single Exposure to Hydrochloric Acid

**DOI:** 10.3390/ijms22168833

**Published:** 2021-08-17

**Authors:** Ruben M. L. Colunga Biancatelli, Pavel Solopov, Christiana Dimitropoulou, John D. Catravas

**Affiliations:** 1 Frank Reidy Research Center for Bioelectrics, Old Dominion University, Norfolk, VA 23509, USA; psolopov@odu.edu (P.S.); cdimitro@odu.edu (C.D.); jcatrava@odu.edu (J.D.C.); 2 School of Medical Diagnostic & Translational Sciences, College of Health Sciences, Old Dominion University, Norfolk, VA 23509, USA

**Keywords:** hydrochloric acid, pulmonary fibrosis, children, TGF-β, heat shock proteins, inflammasome NLRP3

## Abstract

Exposure to hydrochloric acid (HCl) represents a threat to public health. Children may inhale higher doses and develop greater injury because of their smaller airways and faster respiratory rate. We have developed a mouse model of pediatric exposure to HCl by intratracheally instilling p24 mice (mice 24 days old; 8–10 g) with 2 µL/g 0.1 N HCl, and compared the profile of lung injury to that in HCl-instilled adults (10 weeks old; 25–30 g) and their age-matched saline controls. After 30 days, alveolar inflammation was observed with increased proteinosis and mononuclear cells in the bronchoalveolar lavage fluid (BALF) in both HCl-instilled groups. Young p24 animals—but not adults—exhibited higher NLR family pyrin domain containing 3 (NLRP3) inflammasome levels. Increased amounts of Transforming Growth Factor-β (TGF-β) mRNA and its intracellular canonical and non-canonical pathways (p-Smad2 and p-ERK) were found in the lungs of both young and adult HCl-instilled mice. Constitutive age-related differences were observed in the levels of heat shock protein family (HSP70 and HSP90). HCl equally provoked the deposition of collagen and fibronectin; however, significant age-dependent differences were observed in the increase in elastin and tenascin C mRNA. HCl induced pulmonary fibrosis with an increased Ashcroft score, which was higher in adults, and a reduction in alveolar Mean Alveolar Linear Intercept (MALI). Young mice developed increased Newtonian resistance (Rn) and lower PV loops, while adults showed a higher respiratory system resistance and elastance. This data indicate that young p24 mice can suffer long-term complications from a single exposure to HCl, and can develop chronic lung injury characterized by a stronger persistent inflammation and lesser fibrotic pattern, mostly in the airways, differently from adults. Further data are required to characterize HCl time- and dose-dependent injury in young animals and to identify new key-molecular targets.

## 1. Introduction

Hydrochloric acid (HCl) is considered one of the most hazardous chemicals and HCl exposure represents a threat to public health. HCl is widely employed in gas and oil drilling facilities, the steel industry, science laboratories, swimming pool maintenance, and illegal drug manufacturing. Every year, more than ~2.5 million metric tons of HCl are produced and accidental spills have occurred (e.g., in the United States of America, Canada, and China). Exposure to HCl has been associated with both acute and chronic toxicities. Acute exposure to HCl provokes irritation of the skin, eyes, and respiratory tract, and, at high concentrations and prolonged exposure times, can produce acute respiratory distress syndrome (ARDS) and death [1]. HCl fumes are highly soluble, and, in contact with the mucosa of the respiratory tract, produce HCl acid, which causes direct chemical burns to alveolar and vascular tissues [2], acute alveolar hemorrhages [3], and pneumonitis [4]. Even though acute symptoms usually resolve in 3–7 days [5], HCl exposure has been shown to provoke a milder, persistent, and long-lasting inflammatory response, which has been related to the development of chronic lung injuries, including airway hyper-responsiveness [6], reactive airways dysfunction syndrome (RADS) [7], asthma-like conditions [8], and pulmonary fibrosis [9]. In a public health screening of adults and children located within one mile of an HCl accidental spill, persistent abnormalities in spirometry were observed even ten months after the incident in more than 67% of the subjects [10]. Furthermore, abnormal respiratory function was found in almost 30% of participants who did not report any breathing problems, suggesting that HCl-induced chronic lung injury may be underreported and that the real incidence of long-lasting complications after HCl exposure may be considerably higher.

Accidental spills of HCl from swimming pool supply containers and transport vehicles have occurred near schools and have resulted in the hospitalization of children (e.g., in Japan and the United States of America). HCl gas is colorless, but in contact with air vapors, it can develop dense white fumes that concentrate at soil levels, putting smaller individuals and children at higher risk. Children possess smaller airways, greater lung surface area relative to their body weight, and a faster respiratory rate, and for these reasons, the CDC estimates that children likely receive larger doses of HCl and thus develop more severe injury [11]. Little is known about the long-term complications of HCl exposure in children.

Children usually display stronger inflammatory responses than adults [12,13], and if exposed to toxic inhalants, they are more prone to develop bronchial hyperresponsiveness, persistent inflammation, asthma-like conditions, and chronic lung injury. Chronic lung injury in children is a serious complication, as it can affect lung and systemic development and cause growth defects. Additionally, chronic therapy with corticosteroids can further worsen growth defects and affect quality of life [14,15,16].

The fibrotic process is a rare condition in children, and it is not clear if HCl exposure results in chronic lung injury like that seen adults. It is thus important to investigate both the mechanisms of, and the potential countermeasures against, HCl-induced toxicity in this vulnerable population.

We have previously characterized the long-term profile of HCl induced chronic lung injury in adult mice [9], identified sex-related molecular targets [17], and investigated potential countermeasures [18]. Here, we developed a pediatric murine model of HCl exposure by intratracheally instilling HCl in young mice, and, 30 days later, investigated the histological, molecular, and functional differences among similarly treated adults and age matched saline-instilled controls.

## 2. Results

### 2.1. HCl Instillation Provokes Persistent Alveolar Inflammation

**Figure 1:**
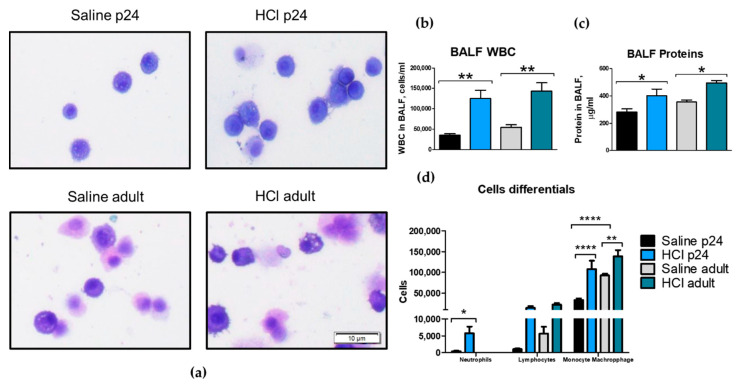
Alveolar inflammation persists in both young and adult mice. (**a**) Microscopic evaluation of bronchoalveolar lavage fluid (BALF) cells revealed monocyte−macrophage infiltration. (**b**) Total cell count, (**c**) total protein concentration, and (**d**) cell differentials. Young p24 and adult mice were instilled with 2 µL/g 0.1 N HCl. Original magnification 100×, scale bars 10 µM. Means ± SEM, n = 3–5, * *p* < 0.05; ** *p* < 0.01; **** *p* < 0.0001 with one-way ANOVA and Tukey’s test.

Exposure to 0.1 N HCl elicited a persistent alveolar inflammation that lasted at least until day 30 post-instillation in both p24 and adult mice. Bronchoalveolar lavage fluid (BALF) displayed increased cellularity and proteinosis for both young p24 and adult mice when compared with their corresponding saline controls (Figure 1a–c). A significant increase in monocytes/macrophages was observed in both age groups 30 days after HCl-instillation, with young animals also displaying increases in neutrophils (Figure 1d).

### 2.2. Histological Evidence of Inflammation and Age-Dependent Activation of NLRP3 Inflammasome

**Figure 2:**
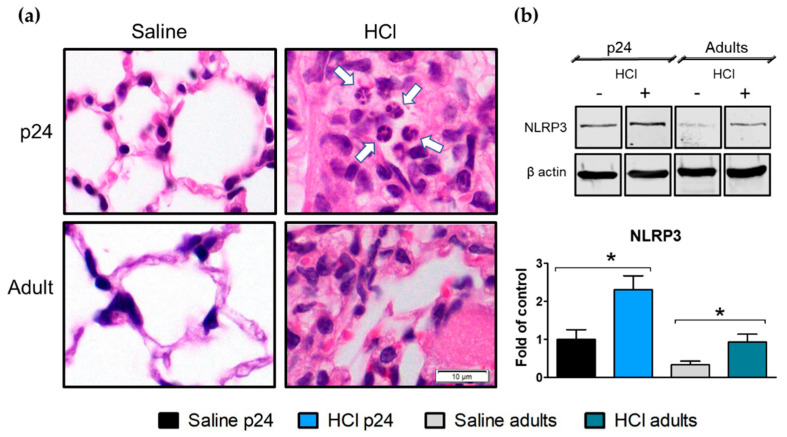
Histological evidence of inflammation and age-dependent activation of NLRP3 inflammasome, 30 days after HCl instillation. (**a**) Hematoxylin and Eosin staining revealed edema; increased alveolar thickness; mononuclear cell infiltration; and, in young HCl-instilled mice, neutrophil infiltration (white arrows). (**b**) Expression of the inflammasome NLRP3 increased in young and in adult mice instilled with HCl. Histograms represent the quantification of the blots. Original magnification 100×, scale bars 10 µM. Means ± SEM, n = 3, * *p* < 0.05; with one-way ANOVA and Tukey’s test.

Thirty days post HCl instillation, severe changes in the lung parenchyma were observed in both young and adult mice when compared with the saline-instilled controls. HCl-treated mice displayed increased cellularity, the presence of hyaline membranes, interstitial edema, and the presence of alveolar and interstitial monocytes−macrophages; additionally, young p24 mice exhibited neutrophil infiltration (Figure 2a). HCl-induced activation of the inflammasome NLRP3 was observed in both young and adult animals (Figure 2b).

### 2.3. HCl Induces Age-Dependent Increases in TGF-β and Its Intracellular Signaling

**Figure 3:**
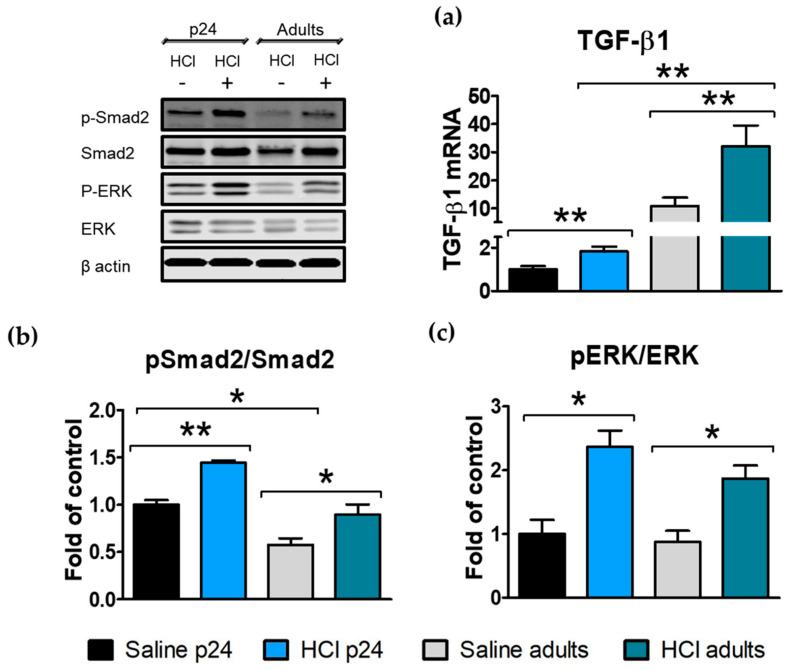
HCl induces age-dependent increases in TGF-β and its intracellular signaling 30 days after HCl-instillation. (**a**) Young and adult mice treated with HCl showed increased expression levels of TGF-β1 mRNA compared with their corresponding saline controls. (**b**) The phosphorylation of Smad2 was significantly increased in both adult and young HCl-treated mice. (**c**) The activation of ERK increased in both young p24 and adult mice instilled with HCl. Histograms represent the quantification of the blots. Means ± SEM, n = 3–4, * *p* < 0.05; ** *p* < 0.01 with one-way ANOVA and Tukey’s test.

Transforming growth factor β (TGF-β) is the leading cytokine in the fibrotic process and plays a crucial role in chronic lung injury. TGF-β mediates epithelial to mesenchymal transformation (EMT) via its canonical and non-canonical signaling pathways [19]. Thus, we measured the lung levels of TGF-β1, and the activation (phosphorylation) of extracellular signal-regulated kinases (ERK) and Smad2 in young (p24) and adult mice instilled with HCl and in age-matched saline-instilled controls. Thirty days after HCl instillation, increased levels of TGF-β1 were still present in the young and adult HCl-instilled animals, with the adults showing the highest values (Figure 3a). The canonical Smad2 and non-canonical ERK pathways of TGF-β1 signaling displayed increased values for both HCl instilled groups, with the adult controls having a constitutively lower expression of phospho-Smad2 compared with the young mice (Figure 3b,c).

### 2.4. Age-Dependent Expression of Heat Shock Proteins in the Lung

**Figure 4:**
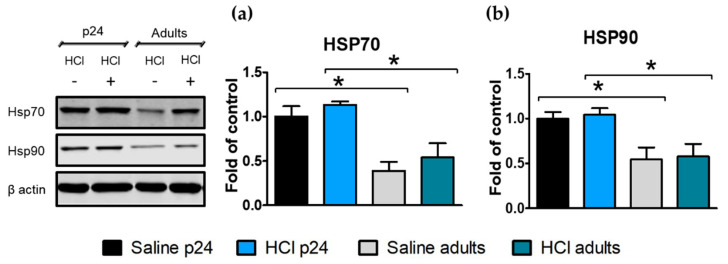
Age-dependent expression of heat shock proteins in the lung. (**a**,**b**) Young control mice express higher concentrations of HSP70 and HSP90 than the adult control mice. No differences were observed 30 days after HCl instillation. Histograms represent the quantification of the blots. Means ± SEM, n = 3–4, * *p* < 0.05; with one-way ANOVA and Tukey’s test.

Heat shock proteins (HSP) are actively involved in protein stabilization and thus play an important role during development. Both HSP70 and HSP90 levels were significantly higher in young p24 compared with adult mice, both saline- and HCl-treated. There were no significant effects of HCl in either age group (Figure 4a,b).

### 2.5. Age-Related Differences in Pulmonary Fibrosis, Ashcroft Score and Mean Alveolar Linear Intercept

**Figure 5:**
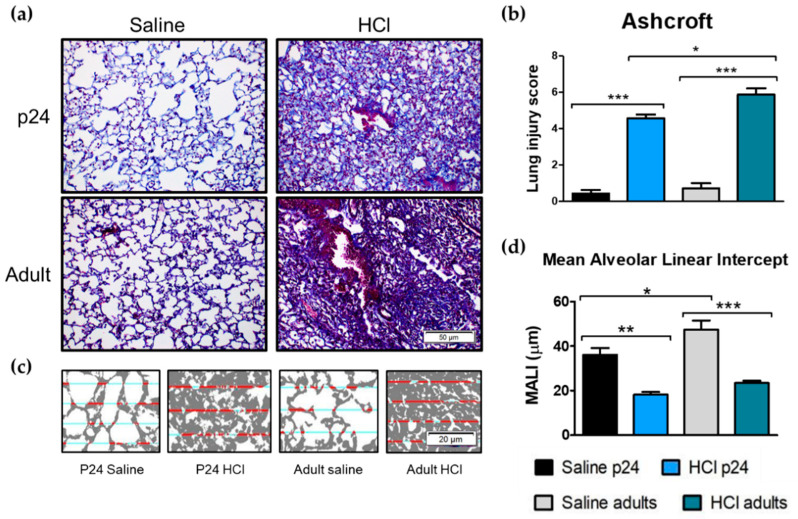
Age-related differences in pulmonary fibrosis, Ashcroft score, and Mean Alveolar Linear Intercept (MALI) 30 days after intratracheal instillation of saline or HCl in young p24 and adult mice. (**a**) Adult mice instilled with HCl display a significant loss of parenchymal architecture compared with the adult controls. Young p24 mice similarly display a fibrotic process with the formation of collagen fibers, but with the maintenance of alveoli and parenchymal structures. Original magnification 20×, scale bars 50 µM. (**b**) Adult mice exhibit higher Ashcroft scores compared with young mice after HCl instillation. (**c**,**d**) MALI is reduced in both young and adult mice. Original magnification 40×, scale bars 20 µM. Means ± SEM, n = 3–5, * *p* < 0.05; ** *p* < 0.01, ***: *p* < 0.001 with one-way ANOVA and Tukey’s test.

Thirty days after HCl instillation, severe histological abnormalities were observed in the lungs of the adult and young mice. Adult mice showed a loss of parenchymal architecture, increased collagen deposition, and large fibrous masses that were more pronounced than those observed in the young mice (Figure 5a). Indeed, the Ashcroft score displayed higher values in HCl-treated adult mice compared with the saline matched controls and HCl-instilled young mice (Figure 5b). Additionally, the mean linear alveolar intercept (MALI; computed with the semi-automated methods as proposed recently [20]) decreased in both adult and young HCl-treated mice, reflecting a loss of alveoli. Notably, the adult controls displayed higher MALI values than the young controls (Figure 5d).

### 2.6. Age-Related Differences in Extracellular Matrix Protein Deposition

**Figure 6:**
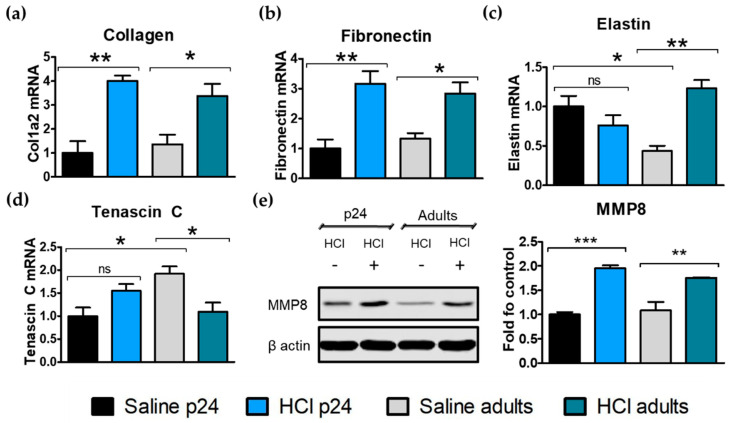
Age-related differences in the extracellular matrix protein deposition. (**a**) Collagen 1a2; (**b**) fibronectin; (**c**) elastin and (**d**) tenascin C mRNA expression levels in the lung computed by real-time PCR. (**e**) Matrix metalloproteinase-8 (MMP8) protein expression estimated by immunoblotting and normalized to β-actin. Means ± SEM, n = 3–5; ns—non-significant; * *p* < 0.05; ** *p* < 0.01, *** *p* < 0.001 with one-way ANOVA and Tukey’s test.

The expression levels of extracellular matrix (ECM) proteins, such as fibronectin, tenascin C, elastin, and collagen, are known to participate actively in the fibrotic process in the lung and to correlate with disease severity and progression. We analyzed ECM mRNA expression levels in young and adult mice 30 days after HCl-instillation. Exposure to HCl significantly increased collagen expression levels in young and adult mice when compared with the controls (Figure 6a). A similar effect was observed for fibronectin, a crucial protein related to the structure of the parenchyma and involved in chronic lung injury (Figure 6b). Elastin expression decreased in young HCl-treated animals but increased in adults after HCl-instillation. The constitutive expression of elastin was significantly higher in the young mice compared with adult mice (Figure 6c). Young p24 mice exhibited a significantly lower expression of constitutive tenascin C compared with the adults, and an increase 30 days post-HCl instillation (Figure 6d). We previously characterized the time-activation of tenascin C, which, in adults, is higher in the early pro-fibrotic stages [9]. Additionally, both young and adult mice exhibited increased levels of matrix metalloproteinase 8 (MMP8) protein expression in response to HCl, an enzyme correlated to the fibrotic process and ECM remodeling (Figure 6e).

### 2.7. Lung Mechanics

**Figure 7:**
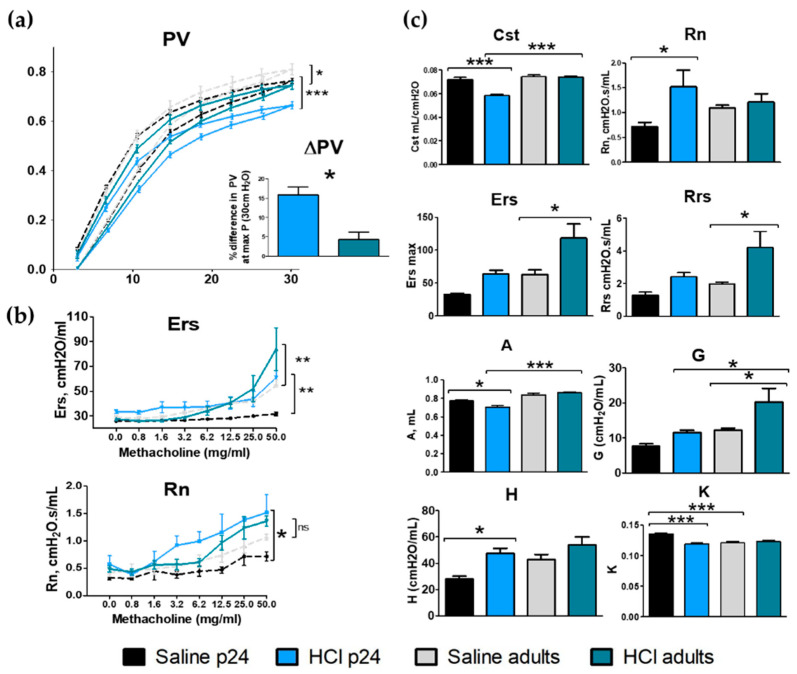
Age-dependent changes in lung mechanics after HCl instillation. (**a**) HCl provokes a downward shift in PV loops, which becomes maximal in young animals. (**b**) Respiratory system elastance (Ers) and Newtonian resistance (Rn) in response to increasing concentrations of aerosolized methacholine in vehicle and HCl-treated young and adult mice. (**c**) Static compliance (Cst), Newtonian resistance (Rn), respiratory system elastance (Ers), respiratory system resistance (Rrs), inspiratory capacity (A), tissue damping (G), tissue elastance (H), and curvature of the PV loops (K) in young and adult mice treated with either HCl or vehicle. Means ± SEM; n = 3–5, * *p* < 0.05; ** *p* < 0.01; *** *p* < 0.001 with one- or two-way ANOVA with Tuckey’s test.

Changes in lung mechanics were analyzed 30 days after HCl instillation in young p24 and adult mice. Both adult and young mice displayed significant changes in pressure volume (PV) loops compared with their corresponding saline controls, with young animals displaying the highest downward shift in PV loops after HCl instillation, both in absolute values and as a percentage of their basal values (Figure 7a). HCl-treated adult and young mice displayed increased values of respiratory system elastance (Ers) in response to methacholine, suggesting airway hyperresponsiveness, while only young mice exhibited increased values of Newtonian resistance (Rn) (Figure 7b). HCl-treated adult mice displayed increased basal values of total respiratory resistance (Rrs), tissue damping (G), and respiratory system elastance (Ers), while young mice showed a reduction in static compliance (Cst), curvature of the PV loops (K), and inspiratory capacity (A), and increased Newtonian resistance (Rn) and tissue elastance (H) (Figure 7c).

## 3. Discussion

We tested the hypothesis that exposure to HCl would provoke chronic lung injury in a pediatric murine model. HCl was instilled intratracheally, as described in [18]; the same dose was administered to adult and young p24 mice (2 µL/g). Even though the CDC considers children to be at a greater risk for chemical exposure—because of their faster respiratory rate, smaller airways, and stronger inflammatory response—there is a lack of evidence regarding pediatric exposure to chemicals [11].

As explained in Methods 4.4, the dose utilized in this study corresponds to adult humans and children exposed for 5 min to 2000 ppm HCl. Our calculations confirm that, due to the physiology of the respiratory tract during development, children exposed for the same time to the same gas concentration, would result in a slighter higher deposited dose than adults.

We employed mice 24 days old (p24), as this age corresponds to a critical phase of post-natal lung development. Indeed, after the initial vascular maturation from days 14 to 21 [21], new alveoli and septa are formed following the preexisting vascular double layered capillary network, and by day 24, just 40% of the alveolar septa are formed; alveolarization is complete by day 36 [22]. Although human and mouse lung development present some critical differences; p24 mice can be considered as a reasonable model of pre-pubertal children between 6 and 9 years old [23]. Thus, our model investigates how chronic lung injury affects immature lungs during their most critical temporal window, which may result in impaired lung development.

The instillation of HCl in either young or adult mice resulted in a persistent inflammatory response characterized by increased proteinosis and mononuclear cells infiltration (Figure 1). HCl is a strong acid that provokes a direct chemical burn to alveolar structures. HCl caused alveolar hemorrhages when intratracheally instilled [3], and increased the permeability of lung endothelial cells, favoring the migration of inflammatory cells, proteins, and liquid to the interstitium [24].

Histological sections of lungs stained for H&E displayed thickening of the alveoli, hyaline membranes, and edema in both age groups. Interestingly only young p24 mice showed infiltration of neutrophils 30 days post-instillation (Figure 1 and Figure 2). Immune cells of BALF of patients with idiopathic pulmonary fibrosis (IPF) showed an increased production of IL-1β and hyper-inducible NLRP3 activation [25]. Similarly, bleomycin-instilled mice displayed age-dependent activation of the inflammasome, while NLRP3 null mice showed reduced fibrosis when compared with their matched counterparts [26]. In our study, young p24 mice and adults, displayed persistent activation of the inflammasome NLRP3, with the young animals exhibiting the highest values.

HCl provokes a milder and persistent pro-fibrotic response guided by TGF-β. Both young p24 and adult mice instilled with HCl showed increased TGF-β1 mRNA in the lung tissue (Figure 3), as well as activation of the intracellular pathways of TGF-β signaling with increased phosphorylation of Smad2 and ERK. Both TGF-β and MAPK/ERK play a role in NLRP3 assembly [27,28]. Additionally, NLRP3 promotes Smad signaling and amplifies the effects of TGF-β [29]. The higher level of NLRP3 activation, observed in our young animals, could have participated in high phospho-Smad2 levels, and thus provoke fibrosis even with relatively lower levels of TGF-β than adults.

HSP70 and HSP90 are chaperones involved in protein stabilization and in the development of the fibrotic process [30]. Specifically, HSP90 sustains key pathways involved in cancer [31], inflammation [32], and fibrosis [19,33]. HSP90 inhibitors promote client proteins ubiquitination and proteasomal degradation, leading to significant amelioration of pulmonary fibrosis, as reported by us and others [18,34]. Heat shock 60, 70, and 90 participate in cell growth and differentiation, and engage postnatal brain and kidney development in rats [35]. In our study, HSP70 and HSP90 showed a constitutive difference between young and adult mice (Figure 4). HSPs increments in young animals, may have been related to the increased requirements of protein synthesis—and thus, their stabilizer chaperones—necessary for alveolar differentiation, septal formation, and lung development.

Pulmonary fibrosis (PF) in children is a rare and heterogeneous condition named children’s interstitial lung disease (chILD) [36]. Children exhibit more inflammatory cells, less fibroblasts, and lower ECM deposition than adults [37]. This fibrosing process is characterized by endoplasmic reticulum (ER) stress without its evolution towards ECM accumulation, alveolar collapse, and re-epithelialization that usually characterize the wound-healing defects observed in adults [38]. Aged mice (35 weeks) showed higher basal and wound-induced levels of TGF-β when compared with adults (8 weeks), which are related to the slowing of wound-healing process and the deposition of a fibrotic scar [39]. Accordingly, we observed that young animals develop lower fibrosis and Ashcroft scores compared with adults (Figure 5).

TGF-β is crucial for lung growth in neonates and children; however, excessive concentrations can undermine post-natal lung development [40] by reducing the expression of phospholipids; surfactant proteins A, B, and C synthesis [41,42,43]; and by inhibiting alveolar branch morphogenesis [44]. Neonatal mice exposed to bromine develop a persistent injury that permanently affects lung development, gas exchange, and the expression of critical transcriptional factors for lung morphogenesis [45]. Exposure of newborn rats to ozone microparticles results in decreased airway length and diameter development, with persistent structural changes 2 months after exposure [46]. The increased TGF-β levels, observed in p24 mice, could have interfered with bronchial development and provoked the increased resistance of the airways (Rn), not observed in adults (Figure 7).

Elastin is a crucial protein required for alveolar maturation and lung development, and, in adults, is a marker of lung disease severity and progression [47]. The reduced deposition of elastin in young mice after HCl-instillation could represent a crucial difference in how young and adult react during the fibrotic process.

Taken together, these data suggest that while adult mice exposed to HCl develop a pure fibrotic response, with increased tissue damping and total systemic resistance, young animals display a lesser fibrotic process with a particular involvement of the airways, with increased resistance and hyperresponsiveness. This data agree with the literature, as children are less likely to develop fibrosis, but more prone to develop asthmatic conditions, especially during development.

Our study has certain limitations. We have investigated only one time-point (30 days) after the instillation of HCl; it would be of great interest to investigate time-dependent changes in HCl-induced chronic lung injury. A more detailed analysis of the inflammatory response could improve our understanding of how young and adults react to lung injury. The investigation of the changes in the activated (phosphorylated) form of HSP90 and its isoforms could provide additional insight into the mechanisms of fibrosis in the young.

Our study identified important age-related difference in the expression of proteins related to chronic lung injury, lung dysfunction, and lung development. Activation of NLRP3 has been observed in both asthma and fibrosis [25,48], and its inhibition may represent a new target in the management of chronic lung injury. Indeed, NLRP3 inhibition in children could represent an even more beneficial intervention than in adults, because of its stronger activation following HCl exposure.

## 4. Materials and Methods

### 4.1. Materials

HCl ACS grade, methacholine USP grade, RIPA buffer, and protease inhibitor cocktail were obtained from Sigma-Aldrich Corporation (St. Louis, MO, USA). Socumb (pentobarbital) USP grade, Anased (xylazine) USP grade, and Ketaset (ketamine) USP grade were supplied by Henry Schein Animal Health (Pittsburg, PA, USA). The 10% formaldehyde was purchased from Thermo Fisher Scientific (Waltham, MA, USA), the BCA Protein assay kit from Pierce Co. (Rockford, IL, USA), EDTA and Western blot membranes from GE Healthcare (Chicago, IL, USA), TRIzolVR and SuperScriptTMIV VILO reverse transcriptase kit from Invitrogen (Carlsbad, CA, USA), RNeasy Mini Kit from Qiagen (Hilden, Germany), and SYBR Green Master Mix from Applied Biosystems (Carlsbad, CA, USA). All primers used for real time quantitative PCR were purchased from Integrated DNA Technologies, Inc. (Coralville, IA, USA). For SDS-PAGE, ProtoGel (30% acrylamide mix) and TEMED were from National Diagnostics (Atlanta, GA, USA), Tris–HCl buffer from Teknova (Hollister, CA, USA), 10% SDS and ammonium persulfate from Thermo Fisher Scientific, and Protein Dual Color Standards and Tricine Sample Buffer from Bio-Rad Laboratories (Hercules, CA, USA). All of the antibodies were purchased from reputable commercial source and have published immunospecificity data. For the antibodies used in the Western blots, rabbit total and phosphorylated (ERK1/2, HSP90, HSP70, NLRP3, MMP8, P-SMAD2, and SMAD2) were obtained from Cell Signalling Technology, Inc. (Danvers, MA, USA), mouse monoclonal anti-beta actin from Sigma-Aldrich Corporation, and IRDye 800CW Goat anti-rabbit and IRDye 680RD Goat anti-mouse antibodies from LI-COR Biosciences (Lincoln, NE, USA).

### 4.2. Ethical Statement

All of the animal studies were approved by the Old Dominion University IACUC and adhere to the principles of animal experimentation as published by the American Physiological Society, and were carried out in Biosafety Level 2 (BSL-2) and Animal Biosafety Level 2 (ABSL-2) facilities at the Frank Reidy Research Center for Bioelectrics in Norfolk, Virginia.

### 4.3. Animals

Male C57Bl/6J (Jackson Laboratories, Bar Harbor, ME) mice were accommodated in a facility under pathogen free conditions. Young mice (p24—24 days of age, 10–12 g weight) and adult mice (10 weeks old, 24–28 g weight) were divided into four treatment groups: (1) p24 mice that were exposed to the vehicle (saline), (2) p24 mice that were exposed to 0.1 N HCl, (3) adult mice that were exposed to the vehicle (saline), and (4) adult mice that were exposed to 0.1 N HCl.

Mice were intratracheally instilled with vehicle or HCl, and after 30 days were subjected to the collection of bronchoalveolar lavage fluid (BALF), lung tissue protein (Western blotting) and mRNA (real-time qPCR) analyses, evaluation of lung function (FlexiVent), and histological analysis. On day 0, the mice were anesthetized with intraperitoneal (i.p.) injections of xylazine (9 mg/kg) and ketamine (90 mg/kg) for p24 mice and 6 mg/kg and 60 mg/kg for adults, respectively. An i.p. bolus of sterile normal saline (10 µL/g) was given as a pre-emptive fluid resuscitation. After cleaning and disinfecting the surgical field, a small neck skin incision (~1 cm) and separation of the salivary glands was made to visualize the trachea. The mice were suspended vertically from their incisors and a fine (25 G for young and 18 G for adults) plastic catheter was advanced into the trachea (~1.5 cm) in such a way that it could be seen through the walls of the trachea. Freshly prepared HCl solution (groups 2–4) or sterile saline (group 1 and 3) was instilled (2 µL/g body weight) and flushed with 100 µL air. The catheter was withdrawn, the neck incision closed by surgical adhesive Vetbond, and the animals were placed in the ventral position in a small chamber on top of a heating pad under supplemental oxygen (slowly weaned from 100 to 21% O_2_) and observed for the next 4 h for signs of respiratory distress. Mice were returned to their home-cages (five mice/cage) and monitored daily for abnormal physical appearances. On day 30, they were euthanized, and the BALF and lungs were collected for analysis, as described above.

### 4.4. HCl Dose Calculation and Translation to Adult and Pediatric Human Exposure

First, 2 µL/g of 0.1 N HCl was instilled into 10 g p24 mice and 25 g adult mice (20 µL and 50 µL, respectively), which corresponds to a 7.17 mg/kg dose. To properly extrapolate this dose to humans, we used allometric calculations proposed by Phillips [49] in Equation (1):

(1) $$X_{h}=X_{a}{\left(\frac{M_{a1-2}}{M_{h1-2}}\right)}^{\left(1-b\right)}$$

where $X_{h}$ = human drug equivalent dose (in mg/kg), $X_{a}\;$= mouse drug dose (7.17 mg/Kg), $M_{a1}=$ adult mouse body mass (0.025 kg),$\;M_{a2}=$ p24 mouse body mass (0.010 kg), $M_{h1}=$ adult human body mass (70 kg), $M_{h2}=$ human children body mass (20 kg), and b = 0.67. For 0.1 N HCl, the equivalent deposited human dose was computed to 0.52 mg/kg for adults and 0.58 mg/kg for children.

Henderson and Howard summarized several sources of HCl human exposure for time, concentration, and their related effects, and defined “dangerous” exposure as 2000 ppm for 5 min [50]. Then, we calculated the amount of deposited dose in adults and children exposed to HCl using equation 2, as proposed by Forbes et al. [51]:

(2) $$Deposited_{Dose}=\left(C\;\times RMV_{1-2}\;\times D_{1}\;\times IF\;\times DF\right)\;÷BW_{1-2}$$

where C = concentration of HCl in the air (2.98 mg/L), $RMV_{1}$ = respiratory minute volume for adults (6 L/min), $RMV_{2}$ = respiratory minute volume for children (2 L/min); $D_{1}$= duration of exposure (5 min), $BW_{1}$= body weight adults (70 kg), $BW_{2}$ = body weight children (20 kg), IF = inhalable fraction (assumed to be 100% for Mass Median Aerodynamic Diameter (MMAD) less than 3–4 µm, as is the case of HCl), and DF = deposited fraction (assumed to be 0.4 in humans [52]. A 5 min, 2000 ppm HCl exposure was thus computed to a 0.51 mg/kg deposited dose for adults and 0.59 mg/kg for children, which is virtually identical to the 0.1 N HCl dose administered in our preclinical murine model of adult and paediatric exposure to HCl.

### 4.5. Bronchoalveolar Lavage Fluid (BALF) White Blood Cell Number and Total Protein Concentration

BALF was obtained by instilling and withdrawing sterile 1× PBS (1 mL) via a tracheal cannula. The fluid was centrifuged at 2500× *g* for 10 min and the supernatant was aspirated and stored at –80 °C. The cell pellet was re-suspended in 1 mL sterile PBS. The total number of white blood cells (WBC) was determined using a hemocytometer. For differential cell counts, within 1 h after collecting BALF, the samples were mixed gently and the cell suspensions were spun onto glass slides using a Cytospin 4 centrifuge (Thermo Fisher) set at 300 rpm for 10 min. The slides were stained using the May−Grunwald−Giemsa staining protocol (Differential Quick Staining Kit, Electron Microscopy Sciences, Hatfield, PA, USA), and a coverslip was mounted. A minimum of 400 cells were identified and counted under light microscopy (Olympus BX-46, Tokyo, Japan). After the fluid was centrifuged at 2500× *g* for 10 min, the supernatant was collected for the total protein. The total protein concentration was determined using a micro bicinchoninic acid (BCA) Protein Assay Kit according to the manufacturer’s instructions.

### 4.6. Histopathology and Lung Fibrosis Scoring

The mice were sacrificed and the lungs were fixed in an upright position. A small transverse incision was made in the middle of the trachea, and the lungs were instilled and inflated with 10% formaldehyde solution to a pressure of 15 cm H_2_O using a 20 G catheter. The trachea was then ligated with sutures; the lungs were removed from the thorax and placed in 10% formaldehyde solution for 72 h. Mid-transverse slices were made from the formalin-fixed lung tissue samples and were embedded in paraffin. Sections 5-µm thick were prepared from the blocks and stained with Masson’s trichrome stain and H&E. Ten randomly selected fields from each slide were examined under 10× and 20× magnification. All of the slides were scored according to the Ashcroft score method in order to estimate the severity of pulmonary fibrosis [53]. The observer was blinded to the treatment.

### 4.7. MeanAlveolar Linear Intercept

The mean alveolar linear intercept (MALI) was calculated utilizing the semi-automated methods recently described [20]. Histological images stained with Masson’s trichome were transformed in eight-bit images and a Huang filter was applied utilizing Fiji ImageJ (https://imagej.net/software/fiji/; accessed on June 2021). An image of the same size with 20 horizontal lines was created and overlaid with 50% opacity. Colour thresholds were then adapted to visualize the intercept between alveolar structures and the grid. The length of the segments was quantified as pixels and scaled to microns (µm). Five images were utilized per group, each one was characterized by the analysis of 800–1000 chords. Data were then plotted as the average value per each group.

### 4.8. Tissue Collection

Immediately after euthanasia, the chest was opened, blood was taken out from the heart through the right ventricle, and the pulmonary circulation was flushed out with sterile PBS containing EDTA. The lungs were dissected from the thorax, snap-frozen in liquid nitrogen, and kept at –80 °C for subsequent analysis.

### 4.9. Western Blot Analysis

Proteins in the lung tissue homogenates were extracted from frozen lungs by sonication (50% amplitude, three times for 10 s) in an ice-cold RIPA buffer with added protease inhibitor cocktail (100:1). The protein lysates were gently mixed under agitation for 3 h at 4 °C, and then centrifuged twice at 14,000× *g* for 10 min. The supernatants were gathered, and the total protein concentration was determined using the micro-BCA assay. Equal amounts of proteins from all lysates were used for the Western blot analysis. The samples were first mixed with Tricine Sample Buffer 1:1, boiled for 5 min, and then separated on a 10–12% polyacrylamide SDS gel by electrophoresis. Separated proteins were then transferred to a nitrocellulose membrane, incubated with the appropriate primary antibody, followed by incubation with the secondary antibody, and were detected by digital fluorescence imaging (LI-COR Odyssey CLx, Dallas, TX, USA). Beta-actin was used as the loading control. ImageJ software v.1.8.0 was used to perform densitometric quantification of the bands (http://imagej.nih.gov/ij/ (accessed on June 2021)); National Institutes of Health, Bethesda, MD, USA). For ERK and p-ERK, both bands were quantified together. Membranes were stripped in a stripping buffer 20 min, blocked, and incubated with other primary and secondary antibodies.

### 4.10. RNA Isolation and Quantitative Real-Time PCR (qPCR)

Lung tissue, stored in an RNAlater solution, was dried and homogenized in TRIzol^®^, followed by a cleaning up step using the RNeasy Mini Kit. The purified RNA was transcribed into cDNA using the SuperScript^TM^ IV VILO Reverse transcriptase Kit and was analysed by real-time qPCR with SYBR Green Master Mix on a StepOne Plus Real-Time PCR System (Applied Biosystems v.2.3). The results were evaluated using the standard curve method and were expressed as the fold of the control values, normalized to β-actin. Specifically designed primer pairs and qPCR conditions were applied to selectively determine the expression of mouse β-actin, TGF-β1, collagen 1α2, fibronectin, elastin, and tenascin C, as previously described [54,55].

### 4.11. Lung Mechanics Measurements

Mice from all groups were anesthetized with pentobarbital (90 mg/kg, i.p.), tracheostomized with a metal 1.2 mm (internal diameter) cannula, and connected to a FlexiVent small animal ventilator (SCIREQ Inc., Montreal, QC, Canada). Ventilation was performed at a tidal volume of 10 mL/kg and respiratory rate of 150/min. A 15-min stabilization period was allowed before any measurements began. Firstly, following a deep inflation, resting static compliance (Cst, mean of three values) and pressure−volume relationships (PV curves) were estimated by stepwise increasing the airway pressure to 30 cm H_2_O and then reversing the process. Both parameters reflect the intrinsic elasticity of the lungs and are either reduced (Cst) or shifted to the right (PV curves) in fibrosis. Secondly, Snapshot-150 and Quick Prime-3 manoeuvres were performed. Respiratory system resistance (Rrs) and elastance (Ers), reflecting the behaviour of the entire respiratory system (peripheral and conducting airways, chest wall, and parenchyma); Newtonian resistance (Rn); tissue damping (G); inspiratory capacity (A); and the curvature of the PV loops (K) reflecting resistance of the large, conducting airways, parenchymal stiffness, and changes in inspiratory gas dynamics, were calculated, and are presented as a mean of 12 recordings.

### 4.12. Statistical Analysis

The results are given as means ± standard error of the mean. Statistical significance of the differences among groups was determined by one-or two-way analysis of variance (ANOVA), followed by the Tukey’s post-hoc test. Statistical analysis was done using GraphPad Prism Software (GraphPad Software, San Diego, CA, USA). The significance level was set at 0.05.

## 5. Conclusions

Hydrochloric acid is a dangerous chemical with acute and chronic toxicity. Age-dependent differences in HCl-induced chronic lung injury were investigated, and higher lung inflammation with increased levels of NLRP3 in p24 young mice compared with HCl-adults was revealed. Young and adult mice alike exhibited activation of TGF-β and its intracellular pathways (p-ERK and p-SMAD2); however, less fibrosis was observed in the young. Age-related differences were observed in the pathophysiological deposition of ECM and while adult mice developed a pure fibrotic process, the young mice showed a mixed pattern with asthma-like conditions and increased airways resistance. Our initial data support the further investigation for HCl toxicity in children and the development of potential countermeasures.

## Data Availability

Derived data supporting the findings of this study are available from the corresponding author upon request.
